# Pathobionts: mechanisms of survival, expansion, and interaction with host with a focus on *Clostridioides difficile*

**DOI:** 10.1080/19490976.2021.1979882

**Published:** 2021-11-01

**Authors:** Harish Chandra, Krishna Kant Sharma, Olli H. Tuovinen, Xingmin Sun, Pratyoosh Shukla

**Affiliations:** a Department of Environmental Microbiology, School of Earth and Environmental Sciences, Babasaheb Bhimrao Ambedkar University, Lucknow, Uttar Pradesh, India; b Department of Molecular Genetics, Biochemistry and Microbiology, University of Cincinnati College of Medicine, Cincinnati, OH, USA; c Laboratory of Enzymology and Recombinant DNA Technology, Department of Microbiology, Maharshi Dayanand University, Rohtak, Haryana, India; d Department of Microbiology, Ohio State University, Columbus, OH, USA; e Department of Molecular Medicine, Morsani College of Medicine, University of South Florida, Tampa, FL, USA; f School of Biotechnology, Institute of Science, Banaras Hindu University, Varanasi, India; g Enzyme Technology and Protein Bioinformatics Laboratory, Department of Microbiology, Maharshi Dayanand University, Rohtak, Haryana, India

**Keywords:** Dysbiosis, toxins, inflammasome, pseudomembranous colitis, inflammatory bowel disease, innate lymphoid cells

## Abstract

Pathobionts are opportunistic microbes that emerge as a result of perturbations in the healthy microbiome due to complex interactions of various genetic, exposomal, microbial, and host factors that lead to their selection and expansion. Their proliferations can aggravate inflammatory manifestations, trigger autoimmune diseases, and lead to severe life-threatening conditions. Current surge in microbiome research is unwinding these complex interplays between disease development and protection against pathobionts. This review summarizes the current knowledge of pathobiont emergence with a focus on *Clostridioides difficile* and the recent findings on the roles of immune cells such as iTreg cells, Th17 cells, innate lymphoid cells, and cytokines in protection against pathobionts. The review calls for adoption of innovative tools and cutting-edge technologies in clinical diagnostics and therapeutics to provide insights in identification and quantification of pathobionts.

## Introduction

Microbes are ubiquitous and abundant forms of life in nature that colonize all forms of life including humans. Microbes have co-evolved and have made niche within the entire human body such as skin, oral and nasal cavities, lungs, urogenital tracts, and gut. A human body in average carries about 10^14^ microbial cells comprising fungi, protozoans, other eukaryotes, and prokaryotes.^1^ Majority of these microbes are present in the gastrointestinal (GI) tract, constituting about 10 times the number of host cells and harboring at least 1000 different microbial species.^2^ Gut microbes live in close interactions with each other as biofilms or as free-living forms in the gut linings of mucus layer. Most are commensal bacteria that live in a symbiotic relationship with the host. Gut microbiota starts to form right from the birth and is critical for shaping the immune system and maintenance of the host health. These commensals play important roles in the host physiology and the development of the mucosal immunity. Further, the tri-directional relationships between gut microbiome, intestinal epithelium, and mucosal immune system contribute to the pathogenesis of gut-related disorders.^3^ Eventually, it leads to changes in the gut microbiome that comprises bacteria, fungi, protozoa, and viruses present as commensals, symbionts, and pathobionts. Microbiomes form dynamic and interactive micro-ecosystems that integrate with macro-ecosystems, including eukaryotic hosts. The gut microbiome profiling using multi-omics techniques, coupled with advanced system biology approaches, have been used to define the “protective microbiome” or “pathobionts.”^4^

The GI tract harbors predominantly members of the Firmicutes, Bacteroidetes, and Actinobacteria phyla.^5^ The human microbiome contributes about 100-fold more genes over the human genome that empower the host with enzymes for metabolism and energy needs such as biosynthesis of vitamins, digestion of polysaccharides, and degradation of mucus.^6^ The microbiome helps maintain integrity of the epithelial barrier and prevent pathogen colonization.^7^ The interactions of the components of the microbiota with the mucosal immune system are highly dynamic and is critical for the maintenance of the epithelial barrier and the sensitization of the mucosal innate immune cells for developing immune tolerance against the components of the microbiota.^8^ However, repeated exposure of certain groups of microbes in the microbiota, known as pathobionts, may result in damage to the host epithelial barrier by inducing exacerbated innate inflammatory response via activation of pro-inflammatory cytokines, inflammasome, induction of specific T_H_17 T cells, and recruitment of neutrophils to the site of infections.^9^ These microbes become pathobionts under certain conditions of endogenous and exogenous factors that alter the gut microbiota contributions to the pathological conditions in the gut. For example, *Helicobacter hepaticus*, a component of murine gut, is implicated in large bowl disease in immunocompromised mice, while the disease is not manifested in healthy mice.^10^

Pathobionts are generally innocuous, but may revert to pathogenic phenotype under adverse conditions.^11^ Proliferation of pathobionts depends on the imbalance in the homeostasis of the symbiotic communities caused by host genetic factors and on shifts in the diet or excessive use of antibiotics and other medications, which lead to dysbiosis.^12^ The GI tract harbors important pathobionts (Table 1) such as *Clostridioides* (formerly *Clostridium*) *difficile* (*C. difficile), H. hepaticus, Helicobacter pylori*, segmented filamentous bacteria (SFB), invasive *Escherichia coli, Proteus mirabilis, Klebsiella pneumoniae, Prevotellaceae* and TM7, and vancomycin-resistant *Enterococcus* spp. These bacteria are involved in various pathological conditions such as Crohn’s disease and ulcerative colitis, collectively called inflammatory bowel disease (IBD) in the GI tract, leading to inflammation and severe colitis.^19^ Ulcerative colitis starts in the colonic region whereas Crohn’s disease can appear in any segment of the GI tract. The exact etiology of IBD is poorly understood; however, bacteriological analysis of stool and gut biopsy samples show that several pathobionts are associated with IBD (Table 1).^28^

**Table 1.** Examples of pathobionts and inflammatory conditions

Gut Pathobionts	Inflammatory condition	Mechanism	References
*Bacteroides fragilis*	IBD and colon cancer	Presence of antibiotic resistance genes *cepA, cfiA*, and *nim*(*A-E*) and *B. fragilis* toxin-mediated pro-carcinogenesis inflammation	^13^
Adherent Invasive *E. coli* (AIEC)	Crohn’s disease	Genetic susceptibility in NOD2, mutation in autophagy genes *ATG16L1* and *IRGM*, and dysbiosis	^14,15^
*Enterococcus faecalis*	Ulcerative colitis	Dysbiosis and inflammation in IL-10 deficient mice	^16^
*Fusobacterium nucleatum*	IBD and colorectal cancer	Dysbiosis and chronic inflammation	^17^
*Clostridioides difficile*	Pseudomembranous colitis	Secretion of toxins TcdA and TcdB mediates disruption of epithelial barrier	^18^
*Helicobacter hepaticus*	Colitis and colon cancer in immunocompromised mice	Th17 mediated inflammation	^19^
Segmented filamentous bacteria	Colitis and intestinal inflammation in mice	Th17 mediated inflammation	^20^
*Helicobacter pylori*	Peptic ulcer disease and gastritis	Type IV secreted CagA protein mediated inflammation in host	^21^
*Proteus mirabilis*	Crohn’s disease	Intestinal inflammation through IL-18, Il-1α and NOD-like receptor signaling pathway	^22^
*Klebsiella pneumoniae*	Colitis in mice; primary sclerosing cholangitis	Pathobiont mediated disruption of epithelial barrier and Th17 mediated inflammation	^23^
*Prevotellaceae* and TM7	Murine colitis	Perturbation in NLRP6 inflammasome pathway	^24^
Vancomycin-resistant *Enterococcus spp*.	Blood stream infection following proliferation in the gut	Broad spectrum antibiotic use downregulates RegIIIγ lectin that kills Gram+ bacteria	^25^
*Citrobacter rodentium*	Murine colitis	Activation of signaling pathways by proteins secreted by type III secretion system	^26^
*Klebsiella oxytoca*	Antibiotic-associated hemorrhagic colitis	Enterotoxins tilimycin and tilivalline mediate DNA adducts	^27^

One of the recent pathobionts that has gained attention is *C. difficile*, which is associated with nosocomial infections that cause mild to severe pseudomembranous colitis.^9^ *C. difficile* infections are nosocomial, but other environmental resources such as soil, water, and domesticated animals also contribute to one-third of the cases.^29^ A healthy microbiota generally provides resistance to *C. difficile* pathogenicity. However, a dysbiotic microbiota triggers the proliferation and subsequent disease pathology.^16^ *C. difficile* is a Gram-positive, spore-forming obligate anaerobe and forms biofilms that communicate through quorum sensing. Pathogenesis of *C. difficile* pathobiont initiates with its spore germination to vegetative cells, which proliferate in the GI tract and produce toxins that lead to severe pseudomembranous colitis via disruption of intestinal epithelial barrier.^30^

In this review, we explore the emergence, expansion, survival, and selection mechanisms of these pathobionts that lead to severe inflammatory diseases in the gut. In addition, the review explores the recent findings on the roles of immune cells such as iT_reg_  cells, Th17 cells, and innate lymphoid cells, and induction of cytokines IL-1β, IL-22, IL-23, and IL-33 in the host for protection against pathobionts. We also highlight the recent tools and technologies that aid in the identification of these pathobionts.

## Emergence of pathobionts

### Pathobiont emergence and the dysbiotic gut

Selection of pathobiont in the gut is a complex and multi-factored process. Emergence and selection of pathobionts leading to chronic inflammatory conditions in the gut results from the perturbations in the enteric microbiota or dysregulation of the tolerant immune system.^8,11,12,31^ Pathobionts under certain compromised physiological conditions in the host may find a favorable environment that selects for a specific group or species of bacteria in the enteric microbiota, leading to their booming growth and proliferation. For example, antibiotic-mediated dysbiosis may select for *C. difficile*, thus leading to infection, while normally abundant Ruminococcaceae and Lachnospiraceae families and butyrate-producers are depleted.^32^ The selected and expanded pathobiont population, due to its high microbial antigenic load and secretions of the virulence factors and toxins, may breach the threshold tolerance of the immune system and integrity of the epithelial barrier.^33^

**Figure 1.**
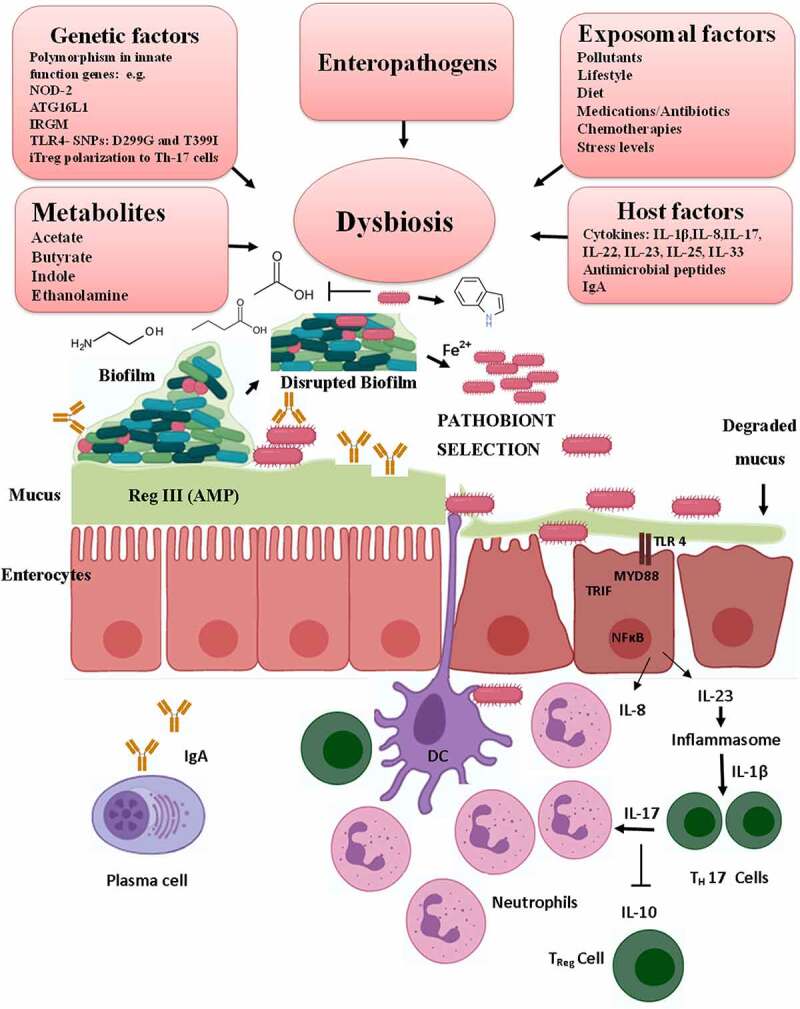
**Pathobiont selection and expansion in the gut by multiple strategies**. Selection of pathobiont in the gut depends on multiple factors. The top panel shows various intrinsic, exposomal factors and enteropathogen infection that drive gut dysbiosis and the disruption of the biofilm. Increased presence of mucolytic species in the dysbiotic gut degrades the protective mucus layer of the epithelial barrier, which can then be accessed by the pathobionts. Further, the dysbiotic gut causes changes in the metabolites that favor pathobiont selection. Pathobionts are further selected by preferentially utilizing the metabolites and efficiently scavenging iron that helps in their exuberant growth and proliferation. The *C. difficile* pathobiont, using quorum sensing through *Agr1* signaling, promotes indole-producing bacteria, which favor the growth and expansion of the pathobiont *C. difficile*, which then produces toxins TcdA and TcdB that disrupt the integrity of the epithelial membrane. The exposed epithelial cells recognize the pathobiont by toll-like receptors cause activation of the inflammasome, secrete chemokines/cytokines, and initiate a cascade of inflammatory response that recruits neutrophils and other immune cells via activation of inflammatory Th17 cells to the site of infection. Illustrations were made using Biorender tool

In healthy individuals, the tolerant mucosal immune system of the host ameliorates pathobiont-mediated detrimental effects on the mucosal epithelial barrier. Activation of the mucosal immune system is critical for tolerance against the microbiota, but its dysregulation by pathobiont may exacerbate the gut inflammation. For instance, the pathobiont *C. difficile* uses toxins TcdA and TcdB to target Rho GTPases in intestinal epithelial cells and activates the pyrin inflammasome to trigger IL-1β secretion. This polarizes the protective Th response to T_H_17, leading to IL-17-mediated recruitment of neutrophils to the site of infections and subsequent severe inflammatory colitis (Figure 1).^34^ However, there are intrinsic, extrinsic factors/exposomal factors, host factors, enteropathogen infection and nutritional factors in the pathobiont selection that disturb the homeostasis of the microbe-host interaction (Figure 1).^14,15,35^

### Intrinsic factors

Intrinsic factors generally include changes in the host genetic factors, which affect the gut mucosal immune functions. One of the host genetic factors that helps pathobiont selection was reported using genome-wide association studies in IBD, showing polymorphism of 163 loci related to the function of innate immunity and predisposing them to increased risk of inflammatory colitis.^15^ One such susceptibility gene in Caucasian populations that predisposes them to Crohn’s disease is the nucleotide-binding oligomerization domain 2 (*nod2*) gene encoding a pathogen-recognition receptor. NOD2 binds with both Gram+ and Gram- cell wall component muramyl dipeptide and helps clearance of the pathogen load. Crohn’s disease patients develop inflammation in Peyer’s patches, and invasion by bacteria, specifically adherent-invasive *E. coli* (AIEC) pathobiont, may be involved as *nod2*-/- Knockout (KO) mice show colonization of the pathobiont.^14^ Other susceptibility genes that trigger Crohn’s disease encode an autophagy-related protein 16–1 (ATG16L1) and an immunity-related GTPase M (IRGM) protein that are components of autophagy pathway (Table 1). Autophagy is an important mechanism for intracellular bacterial killing by epithelial cells.^15^

**Figure 2.**
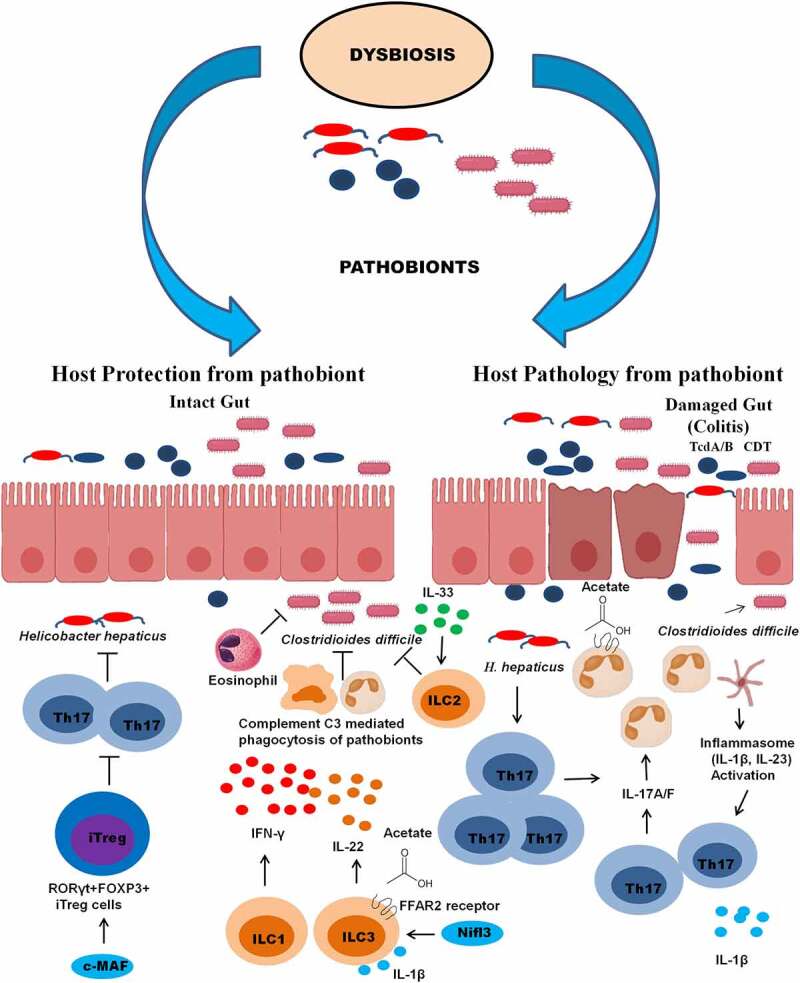
**Host protection and pathology from pathobiont assault**. The induction of protective and pathogenic immune response during infections by pathobionts  such as *C. difficile* and *H. hepaticus*. During pathogenesis both pathobionts produce toxins such as CDT and TcdA/B causing a disintegration of the epithelial barrier. Leaky gut induces an acute inflammatory response mediated by inflammasome activation leading to induction of IL-1β and IL-23 cytokines. The cytokines activate the proliferation and induction of Th17 response leading to neutrophilia and enhanced inflammatory conditions of pseudomembranous colitis and colitis. On the contrary, pathobiont tolerance can be induced by T cell-dependent group of IgA and IgG antibodies against toxins and production of cytokines such as IFN-γ by ILC1s, IL-22 by ILC3s, promoting complement-mediated phagocytosis of pathobiont. In the case of *H. hepaticus*, the expression of c-MAF transcription factor induces the production of pathobiont-specific RORγt+FOXP3+ regulatory T(iT_reg_) cells that confine pro-inflammatory T helper 17 (TH17) cells leading to the tolerance against the pathobiont. IL-33 activates ILC2 that inhibit the proliferation of *C. difficile*. Microbiota-derived acetate interacts with the FFAR2 receptors on ILC3s and neutrophils, leading to their recruitment to the site of infection and activation of inflammasome and secretion of IL-1β. In response to IL-1β, ILC3 further secretes IL-22 through IL-1 receptor signaling. Illustrations were made using Biorender tool

Using a pathogenic strain of *H. hepaticus* in mice, Xu *et al*.^35^ demonstrated that the host induces tolerance against the pathobiont in healthy individuals by expression of RORγt^+^FOXP3^+^ regulatory T (iT_reg_), which restrains the pathogenic T_H_17 cells and is dependent on the expression of the transcription factor c-MAF as discussed in detail in the latter sections of this review (Figure 2).^35^ These results demonstrated the key roles of host genetic factors that drive IBD. The potential role of microbiota-specific or pathobiont-specific virulence factors/metabolites cannot be ruled out, either. Further studies in this direction may help understand how the host factors shift the host tolerance toward pathobiont to pathogenic phenotype.

The tolerance against pathobiont may be further compromised by single nucleotide polymorphism (SNP) in the TLR4 gene expression of the surface receptor, which recognizes lipopolysaccharides on Gram-negative bacteria. Two such SNPs on TLR4 associated with ulcerative colitis are D299G and T399I that prevent the interaction of TLR-4 with the TLR adaptor molecules MYD88 and TRIF^36,37^ (Figure 1). The TLR4 surface receptor is crucial for innate host response as it recognizes Gram-negative bacteria and helps in their elimination. Further experimental studies may reveal the mechanistic details of these SNPs that trigger the expression of inflammatory cytokines upon modulation of TLR4 mediated immune response in ulcerative colitis patients.

### Exposomal and host factors

The exposomal factors are environmental triggers including air pollution, diet patterns, physical activities, stress levels, pathological conditions, antibiotics and other medications, radiation and chemotherapies, and infections in the gut. These exposomal factors greatly influence the homeostasis of the microbe–host equation.^14,15,35^ Other factors include host secretions such as cytokines/chemokines in response to the bacterial arsenals like cell wall components, lipopolysaccharides, flagellar proteins, short-chain fatty acids, and toxins, which facilitate microbe–host interactions, possibly affecting the composition of the gut microbiome and driving inflammatory reactions.

### Enteropathogen infections

It is widely known that microbiota protect against enteropathogen infections. In contrast, how enteropathogens may play a role in dysbiosis of the gut microbiota is poorly understood. Embedded in a matrix of extracellular polymeric substances in the intestinal mucus, gut microbiota predominantly consist of a complex of poly-microbial biofilms, which may disperse free-swimming bacteria as well.^38^ Pathobionts under conditions of a physiological disturbance may slough off from the biofilms, thus leading to inflammation. It has been observed that the inflammatory IBD complications in patients are exacerbated after enteropathogen infections due to dysbiotic gut.^11^ Enteropathogens such as *Giardia duodenalis* perturb the beta diversity of the microbiota and increase the abundance of Firmicutes like Clostridiales by disrupting the microbial biofilm polysaccharide matrix, thereby releasing the pathobiont.^11,38^ There are other enteropathogens like *Campylobacter jejuni* that has been shown to activate latent virulence genes of fimbriae, flagella, and hemolysin E in noninvasive *E. coli*, thus facilitating adherence and translocation through the epithelial barrier.^39^

### Nutritional factors

Pathobionts may have nutritional advantage if they use metabolites that commensal bacteria cannot utilize. For example, the genes in the *eut* operon in the enterohemorrhagic *E. coli* encode enzymes for utilization of ethanolamine, a breakdown product of the intestinal cell wall membrane. This operon is absent in the nonpathogenic *E. coli*.^40^ Iron in the intestinal microenvironment has central role in pathobiont selection and pathobiont-mediated inflammatory conditions and is essential for many biosynthetic process and gene regulation. The host has evolved, therefore, efficient iron acquisition and sequestration systems and hence the free iron available to the microbiota is very limited in the range of 10^−18^ M. Overexpression of *fur* genes related to iron assimilation have been reported in AIEC strain NC101.^41^ This strain produces cellulose, which is a major exopolysaccharide matrix component contributing to iron-induced aggregation. Abrogation of cellulose production reduced the AIEC-mediated colitis in IL10-/- KO mice, which are highly sensitive to inflammation.^41^ Altering the availability of iron modulates the community structure in wild type and in IL10-/- KO mice.^42^ Depletion of the iron in the lumen was linked with dysbiotic states, inducing iron scavenging by Enterobacteriaceae pathobionts.^42^

Pathobionts have also evolved to thrive under iron-limited conditions due to efficient iron scavenging systems. Limited iron availability signals in many pathobionts and pathogens of the GI tract the induction of virulence genes. *C. difficile*, which may progress to infection with a fatal inflammatory condition of pseudomembranous colitis, has been shown to thrive well under iron-limited conditions.^43^ A transcriptional analysis of *C. difficile* under iron-limiting conditions demonstrated the induction of genes related iron assimilation, biosynthesis, and virulence.^44^ In bacteria, available ferrous iron is transported by ferrous iron permeases (Feo transporters) with a membrane-bound unit (FeoB) and a cytoplasmic unit (FeoA). Two of the three Feo transporters (Cd630_14770-14,790 and Cd630_32730-32,740) in *C. difficile* strain 630 were induced under iron starvation conditions caused by 2,2-dipyridyl treatment, an iron chelator.^44^ Many other genes were also induced that are essential for components of polyamine biosynthesis, histidine biosynthesis, motility, and regulation of ferric iron uptake (Fur).^44^

Polyamines have been reported as virulence factors, which help escape immune attacks and play important roles in siderophore uptake and biofilm formation.^45^ It has been reported that polyamines in actively growing Caco-2 cells (human epithelial cell line) can chelate metals and thus co-transport iron.^45^ Therefore, a thorough understanding of the iron assimilatory system of key gut pathobionts may open new therapeutic targets for efficient elimination of the pathobiont and controlling the exacerbated inflammatory response. Activation of iron utilization and transport mechanism in pathobionts under low iron condition may further aggravate the inflammatory colitis.

### Commensal metabolite-mediated pathobiont emergence

Composition of commensals and their metabolites are key to pathobiont selection. Alteration of a specific commensal community impacts the availability of metabolites such as indole and short-chain fatty acids acetate and butyrate, which play crucial roles in the pathobiont selection and expansion. In the last decade, many studies have focused on identification of specific components of the microbiota in both CDI and IBD conditions. Sharp decreases in the abundance of specific commensals in the phylum Verrucomicrobia and the family Leuconostocaceae (lactate- and acetate-producers) in ulcerative colitis patients have been reported.^46^ Reduction of acetate-producers is significant in the selection of a pathobiont as a recent study^47^ showed that acetate is involved in protection from the pathobiont *C. difficile* in the CDI mouse model. Acetate interacts with the free fatty acid receptor two on innate immune cells, helping in inflammasome activation and immune cell recruitment at the site of infection.

The Bacteroides group is involved in mucin degradation during acute inflammatory response in colitis patients.^46^ The depletion of short-chain fatty acids in IBD patients may be due to the low abundance of *Akkermansia muciniphila* from phylum Verrucomicrobia in CDI and IBD patients. *A. muciniphila* is a mucus-degrader and produces fatty acid metabolites such as succinate, acetate, propionate, and 1,2-propanediol ester.^46^ As another example, reduction of butyrate-producers directly affects gut health and inflammation in CDI and IBD.^32^ Butyrate, a fermentative metabolite produced by bacteria in the *Lachnospiraceae* and *Ruminococcaceae* families, is involved in the assembly of epithelial tight junctions, essential for protection of epithelial barrier and immune health.

Metabolic changes in the gut further add complexity by increasing oxidative stress at gut epithelial surfaces, favoring dysbiosis of protective microbiota in the gut. A decrease in the abundance of the group blautia (butyrate-producers) has been reported in exacerbated conditions in CDI and IBD patients. Fecal transplants of the blautia group such as *Blautia obeum* have been shown to restore the healthy state of the gut in CDI patients. Spore germination by *C. difficile* requires bile salts to initiate colonization in the gut, but the production of the bile salts hydrolase by *Blautia* may avert the growth of *C. difficile*.^48^ *B. obeum* strain A2-162 has antimicrobial activity against CDI by producing an antimicrobial peptide nisin that creates unfavorable conditions for the colonization and growth of *C. difficile* in the gut.^49^ There are several bile acid 7α-dehydroxylating gut bacteria such as *Clostridium scindens* and *Clostridium sordellii* that can inhibit the growth of *C. difficile*. These two *Clostridium* species secrete 1-acetyl-β-carboline and turbomycin A, respectively, both of which are the tryptophan-derived antibiotics.^50^ Therefore, fecal microbiota transplantation (FMT) may help in treating recurrent CDI patients. In this regard, promising results have been obtained with at least 68% success rate using fecal transplants from healthy relatives.^51^

Darkoh *et al*.^52^ reported the detection of a significant amount of indole in stool samples of CDI patients. They also showed that the *C. difficile* induces indole-producing microbiota. Quorum sensing properties of microbes is an important aspect of CDI pathogenesis. A mutant of the accessory gene regulator 1 (*agr1*), a component of the quorum sensing system, was defective in inducing indole production, while the complementation of the mutant abrogated the defect and induced indole production. Thus, the antimicrobial antioxidant indole production can prevent the growth of the protective indole-sensitive bacteria and facilitate the expansion and growth of the pathobiont *C. difficile*.^52^

Kang *et al*.^50^ reported that *C. difficile* also secretes proline-based cyclic dipeptides such as cyclo (Phe-Pro) and cyclo (Leu-Pro) that can inhibit the growth of several common gut bacteria including *Staphylococcus aureus* MRSA.^50^ These antimicrobial cyclic dipeptides have been shown to regulate quorum sensing in other groups of bacteria such as *Pseudomonas aeruginosa*.^53^ Thus, *C. difficile* has evolved with biomolecular strategies to inhibit other bacteria in the gut. It is possible that these secretions help *C. difficile* emerge as pathogenic after antibiotic treatments.

## Function and disruption of host intestinal barriers

Intestinal mucosal barrier in the gut is the first line of defense against microbial communities comprised commensals, enteropathogens, and pathobionts.^54^ The intestinal barrier layer consists of different kinds of epithelial cells that are firmly connected with each other by tight junctions. The layer is further covered by mucus secreted by the goblet cells of the host. There are at least six modified epithelial cell types in the intestinal layer with varied functions: goblet cells, tuft cells, Paneth cells, enterocytes, microfold cells, and enteroendocrine cells.^54^ Mucus in the gut is a key physical barrier that protects the host from vast reservoirs of dynamic microbiota. The intestinal mucus layer is an interface that promotes innate immunity to invading pathogens and pathobionts. Sensitized mucus layer contains some immune components like anti-microbial peptides and secretary IgA antibodies.^55^

Mucus is a mucin glycoprotein that consists of *O*-linked and *N*-linked glycans that form a gel-like structure. There are about 20 different types of mucin glycoproteins that are found in humans. Mucin 2 (MUC2) is the predominant form present in the intestines and the colon, and is secreted by the goblet cells.^55,56^ Goblet cells continuously replenish the mucus and keep the gut lubricated with a more dense and rigid layer outside the epithelial cells, which is resistant to bacterial invasion.^54^ However, a less dense and viscous layer is formed outside the firm mucus layer by the mucolytic action of the commensals and the pathobionts. Besides protection of the epithelial barrier, this less dense layer serves as a nutritient source for many microbial communities.^54^ MUC-2 also protects the host from inflammation, and depletion of this mucin may predispose the host to inflammatory conditions. The muc2-/- KO mice have been shown to develop inflammation due to the depletion of the mucin layer.^57^ In this regard, cytokine IL-22 has been shown to be involved in mucin biosynthesis, and IL-22-/- KO mice show exacerbated and lethal conditions of inflammatory colitis.^58^

It is not clear how gut pathobionts interact with mucosal layer and what tools they have evolved to degrade mucosal glycans to cross the underlying intestinal epithelial barrier. Many studies have shown the penetration of intestinal mucus layer by GI pathogens. The pathobiont *C. difficile* has been shown to bind mucin in cell lines and in animal models.^59^ A recent study^60^ found decreased levels of MUC2 in CDI patients with MUC-1 as the primary secreted mucin. The CDI mucus had less *N*-acetyl galactosamine (GalNAc) and elevated levels of *N*-acetyl glucosamine (GlcNAc) and terminal galactose residues.^60^ It has been shown in animals models that the terminal galactose residues act as receptors for *C. difficile* toxin TcdA.^60^ The CotE protein, which is expressed on the surface of *C. difficile* spores, directly binds to mucus with the mucin glycoprotein GlcNAc and GalNAc residues and has mucolytic activity. The absence of CotE has been shown to reduce significantly CDI colonization and virulence in animal models.^61^

Adhesion to the mucosal surfaces through the expression of flagellar proteins in pathobionts provides another opportunity for colonization and pathogenicity. The *C. difficile* flagellar proteins flagellin and flagella cap protein encoded by the *fliC* and *fliD* genes, respectively, have been implicated in *C. difficile* colonization and pathogenicity by adherence to the mucus in a murine study.^59^ Mutant strains that lack flagella components have been implicated in poor adherence to mucus and manifestation of low virulence. In this regard, c-diGMP has been shown to play a vital role in flagellar expression, biofilm formation, and adhesion.^62^ Elevated levels of c-diGMPs have been reported to downregulate flagellar expression and inhibit toxin synthesis and motility via binding to riboswitch upstream of the *flgB* operon.^62^ Additionally, c-diGMP induces the expression of type IV pili that interact with intestinal epithelium cells that facilitate the biofilm formation.^63^ There are other virulence proteins (Spo0A,Cwp66, Cwp84, S-layer protein A, and adhesin fibronectin-binding protein A) that facilitate *C. difficile* adhesion and biofilm formation.^64^ Therefore, upon selection and expansion, the pathobiont can use various tactics such as toxins, cell wall proteins, and flagellar proteins to disrupt the host intestinal barrier, leading to severe colitis in the host.

Another important barrier is the tight junction between the adjacent cells that seals the paracellular pathway, controls the leakage of transport of solutes and water in the epithelial barrier, and facilitates as channels for transport of small cations, anions, or water. Tight junctions are composed of multiprotein complexes and form a network of sealing strands. The major protein complexes are the junctional adhesion molecules, occludins, and claudins.^65^ These proteins in association with peripheral membrane proteins like zonula occluding-1 (ZO-1) anchor the strands to the actin protein of the cytoskeleton and help join the tight junction to the adjacent cytoskeleton of the cells.^66,67^ The junctional adhesion molecules help regulate paracellular pathway and are key for the maintenance of cell polarity. Occludins regulate paracellular permeability and are vital for the integrity of the cell structure and barrier function. Claudins help seal the paracellular space and are important backbone for the tight junctions.^66,67^ Pathobionts and some pathogens disrupt the underlying junctions by secretions of various toxins to destabilize the barrier function. The pathogen *V. cholerae* produces zonula occludens toxin, which interacts with the ZO-1 protein and disrupts the epithelial barrier.^66^ A close relative of the pathobiont *C. difficile, Clostridium perfringens* uses its enterotoxin (CpE) to destabilize the tight junction via claudin protein.^67^

The pathobiont *C. difficile* disrupts the epithelial barrier integrity by disruption of actin cytoskeleton using its toxins TcdA and TcdB.^68^ Activity of these toxins involves four structurally homologous domains: the glucosyl transferase domains, an adjacent cysteine protease domain, a translocation domain, and a receptor-binding domain.^69^ The other two domains contain a hydrophobic sequence and a CROP domain (combined repetitive oligopeptide repeat). Entry into the epithelial cells is facilitated by the CROP domain via cell surface receptors.^69^

TcdA binds with the carbohydrate moieties and GP6 on the apical side of the cells through its CROP domain, which has about 38 repeats. The CROP domain of TcdB has less repeats and has been reported to interact with chondroitin sulfate proteoglycans-4.^69^ Two additional receptors for TcdA, the sulfated glycosaminoglycans and low-density lipoprotein receptor, facilitate the binding of TcdA and entry into the host cells.^70^ The entry of TcdB has been also demonstrated by interaction with poliovirus receptor-like 3 (PVRL3) on the epithelial cells and frizzled protein, a Wint receptor.^71,72^

Binding of TcdB with the frizzled protein receptor also inhibits Wnt signaling, which plays a crucial role in renewal of colonic stem cells and differentiation of colonic epithelium.^72^ Upon entry into host cells, the cysteine protease domain autocatalytically releases glucosyl transferase with the help of inositol hexakisphosphate into the cytosol from the endocytosed endosomes containing the toxin protein. Thereafter, the glucosyl transferase domains in the cytosol inactivate the host Rho GTPases by glucosylation, leading to disruptions of cytoskeleton, tight junction disruption and the epithelial barrier.^71^

TcdB plays more important roles than TcdA in mediating damage to the epithelial barrier function, inflammation, and mortality as TcdB is a more potent toxin than TcdA.^73^ Identified clinical isolates from CDI patients are variably A^−^/B^+^ or A^+^/B^+^ with varying differences in toxicity. These differences in toxicity can be related to the sequence differences between *C. difficile* strains.^74^ In addition to TcdA and TcdB, some hypervirulent strains like the BI/NAP1/027 type carry a binary toxin *C. difficile* transferase (CDT), which consists of proteins CdtA and CdtB.^73^ CDT enters epithelial cells through a lipolysis-stimulated lipoprotein receptor. Upon entry into the cells, CdtB forms pores on the membrane of the endosomes and facilitates the release of CdtA in the cytosol of the cells.^73^ Protein CdtA has ADP-ribosyl transferase activity and ribosylates actin in the cells, interfering with actin polymerization. The action of CdtA contributes to the formation of cell surface protrusions that enhance further adhesion of the *C. difficile* to the host epithelial cells.^73^

## Induction of inflammation in the host

Induction of excessive inflammation by the selected pathobiont is the key factor that drives the inflammatory colitis in the host. The pathobiont *C. difficile* causes inflammation of the colon, also known as pseudomembranous colitis, through the release of TcdA and TcdB. Previous studies^30^ have established that TcdB induces the secretion of pro-inflammatory cytokines such as TNF-α and IL-1β, which activate the mitogen-activated protein kinases (MAPKs) including p38, JNK1/2, and ERK1/2 through NF-κB signaling. Furthermore, TcdB induces the chemokine IL-8 through ERK1/2 signaling.^75^ It has been observed that TcdA-treated cells increased levels of the NF-kappaB homodimers p65/p65 and further enhanced the phosphorylated IkappaB kinase (IKK) alpha/beta and NF-kappaB-inducing kinase (NIK) levels. Additionally, the inhibition of IKK or NIK repressed the upregulation of the interleukin-8 and the monocyte-chemotactic protein-1 target genes of NF-kappaB.^76^

Li *et al*.^77^ reported the role of a MAP kinase phosphatase DUSP1 on inhibition of the MAPK activity. A knockout mutant of TRIM46, which ubiquitinates DUSP1, could inhibit TcdB-induced MAPK and NF-κB signaling, leading to the repression of TNF-α and IL-1β. Furthermore, the mutant inhibited the increased colonic inflammation induced by CDI.^77^ Additionally, the overexpression of the E3-ubiquitin ligase TRIM46 induced elevated levels of proinflammatory cytokines TNF-α and IL-1β. Li *et al*.^77^ also showed that these effects were mediated by binding of the TRIM46 to the promoter region of the NF-κBp65 subunit. In addition to pro-inflammatory mediators in acute colitis of CDI, inflammation was further accompanied by infiltration of immune cells.

The CX3CL1 chemokine has been shown to have chemoattractant activity toward monocytes/macrophages, NK cells, and T cells.^78^ CX3CL1 is upregulated in murine intestinal epithelial cells in response to TcdA, which is dependent on the nuclear factor-kappaB (NF-κB) and IKK activation. Furthermore, p38 MAPK activation was necessary for IKK, and NF-κB activation for induction of the CX3CL1 in the intestinal cells.^78^ Bobo *et al*.^79^ showed the activation of the phosphorylated protein kinase 2, which mediates p38-dependent inflammation in intestinal epithelial cells exposed to TcdA and TcdB. Furthermore, the secretion of IL-8 and GROα was dependent on MK2 activity, and specific inhibition of MK-2 abolished IL-8 secretion. Elevated levels of pMK2 were also found in stools of CDI patients, which suggest the role of this pathway in intestinal inflammation.^79^

In addition to the key role of toxins in mediating inflammatory response, other components such as flagella and secreted membrane vesicles from *C. difficile* have been implicated in inflammation. Flagellin coded by *fliC* has been shown to interact TLR5, which activates NF-κB signaling through MAPK pathways leading to the induction of pro-inflammatory mediators.^80^ *C. difficile* membrane vesicles, which contained about 260 proteins but were devoid of TcdA and TcdB, have been shown to induce pro-inflammatory cytokines such as interleukin IL-1β, IL-6, IL-8, and monocyte chemoattractant protein-1 in human colorectal epithelial Caco-2 cells.^81^ Cytotoxicity of vesicle proteins in Caco-2 cells was also noted.^81^ Identification of the specific membrane vesicle proteins that mediate inflammatory responses may shed further light in the pathogenesis of *C. difficile*.

### Activation of inflammasomes

Inflammasomes are immune system receptors and sensors involved in mediating host inflammatory disorders. Different types of inflammasomes and their assemblies have been reviewed elsewhere.^82^ The important hallmarks for the induction of various inflammasomes are the activation of caspase-1 and the maturation and release of IL-1β and IL-18 cytokines.

Many pathobionts can target Rho GTPases in intestinal epithelial cells to disrupt the homeostasis of the cytoskeleton dynamics.^82,83^ Microtubules of the cytoskeleton play an important role in the activation of the pyrin inflammasome. Cowardin *et al*.^84^ demonstrated that Rho glucosylation by TcdA is important for inflammasome activation by glucosylation-deficient toxins. TcdB can activate pyrin inflammasome, leading to inflammatory cell death known as pyroptosis by caspase-1 activation, which clears the spread of invading pathogen by cell death.^34^ It has been reported^82^ that TcdB-mediated pyrin inflammasome activation is inhibited by colchicine drugs that destabilize microtubules. TcdA and TcdB have been shown to trigger inflammasome activation in macrophages and human mucosal biopsy specimens that induced the IL-1β marker of inflammasome activation.^30^ Furthermore, toxin-induced inflammasome activation and inflammation in ASC-deficient mice were abolished, which were correlated with the pretreatment with anakinra, an IL-1 receptor antagonist that ameliorated the disease condition.^30^ (ASC = apoptosis-associated speck-like protein containing a caspase-recruitment domain.)

Liu *et al*.^85^ demonstrated the ATP-P2X7 pathway of inflammasome activation in macrophages in response to toxigenic *C. difficile* VPI 10463 (*tcdA^+^, tcdB*^+^) infection, leading to pyroptosis by caspase-1 activation. Surface layer proteins from pyroptotic cells were released as result of the *C. difficile* death by inflammasome activation. The response was robust as compared to the non-toxigenic *C. difficile* CCUG 37780 (*tcdA^−^, tcdB*^−^) strain. Additionally, the Ac-YVAD-CMK peptide inhibited caspase-1, which increased the colonic inflammation and bacterial load. The results suggested that this pathway of inflammasome activation plays an important role against bacterial defense and pathogen clearance.^85^

Cowardin *et al*.^86^ reported the secretion of the pro-inflammatory cytokine IL-23 in response to TcdA and TcdB. The secretion was associated with inflammasome activation and was dependent on MyD88-dependent danger signals, pathogen-associated molecular patterns, and host damage-associated molecular patterns. Additionally, IL-23 production was enhanced in the presence of increased IL-1 receptors.^86^ The secretion of IL-23 decreased when inflammasome activation was inhibited in the presence of extracellular K^+^ ions.^86^ In the case of murine model of IBD, dextran sulfate sodium and 2,4,6-trinitrobenzene sulfonic acid-induced colitis NLRP3 inflammasome is activated. Moreover, the *nlrp3*-/- KO mice ameliorated the induced colitis.^87^ Similarly, human colonic biopsy samples from Crohn’s disease and ulcerative colitis showed increased levels of NLRP3, ASC, caspase-1, and IL-1β mRNAs that were associated with exacerbated disease condition.^88^ Thus, the activation of inflammasomes plays a key role in mediating immune responses, which are crucial for host survival. In parallel, however, overstimulation of the inflammasomes exacerbates the inflammatory conditions caused by the pathobionts of the gut.

### Induction of neutrophil response

The innate response is the first line of defense against the pathobiont *C. difficile*. Among the innate cells, neutrophils are the first to mount an antimicrobial response for containment of the pathobionts. An overwhelming response may prove detrimental to the host tissues as it may lead to the exacerbated colitis (Figure 2). Neutrophil infiltration starts at the site of the tissue damage marked by the proinflammatory responses in terms of cytokines/chemokines and generation of reactive oxygen species, the hallmark of the inflammatory condition. In early response to *C. difficile* toxin or spore infection, signaling events are activated, leading to the induction of cytokines/chemokines for the recruitment of the neutrophils.

Multiple studies have shown that the inhibition of chemokines/cytokines such as CXCL1, CXCL2, IL-8, IL-23, IL-17, GM-CSF, MIP2, and leptins leads to reduced neutrophil recruitments to the site of infections.^89–91^ Elevated levels of the granulocyte colony stimulating factor G-CSF at the site of inflammation were reported in the mouse model of CDI and in infected patients. G-CSF helps the egress of mature neutrophils from the bone marrow to the site of inflammation, which requires further interaction from the chemokines CXCL1 and CXCL2 for efficient recruitment of neutrophils.^92^ The granulocyte macrophage colony-stimulating factor GM-CSF has also been reported to have a role in neutrophil influx. *C. difficile*-challenged mice treated with Anti-GM-CSF monoclonal antibodies showed reduced expression of CXCL1 and CXCL2 along with inflammatory cytokines and TNFα, IL-1β, and the inducible nitric oxide synthase.^89^

McDermott *et al*.^90^ showed the role of the inflammatory cytokine IL-23 in neutrophil recruitment using *C. difficile*-infected, p19(-/-) Il-23 deficient mice. They demonstrated that the KO mice were defective in recruitment of CD11b^High^ Ly6G^High^ neutrophils, chemoattractants, and stabilizing factors like CXCL1, CXCL2, CCL3, and CSF3 to the site of infection in the colon. Induction of other inflammatory cytokines such as IL-33, TNF-α, and IL-6 was also reduced in the IL-23-/- KO mice.^90^ Reduced expression of IL-17a and IL-22 cytokines was also observed. However, IL-17a-/- KO mice or anti-IL-22 antibody treated mice did not show abrogation of neutrophil recruitment and inflammatory cytokine induction in the study.^90^ Inflammatory response is critical for the pathology of the CDI and recruitment of neutrophils to the site of infection. Various chemokines and cytokines discussed above play key roles in initiating the inflammation and the recruitment of neutrophils. Therefore, a thorough understanding of their induction and inhibition studies may provide opportunities for development of therapeutic intervention tools for CDI.

In *C. difficile* infection, the nucleotide-binding oligomerization domain-containing protein NOD1, an intracellular pattern recognition molecule, also plays a key role in neutrophil recruitment.^93^ The *nod1-/-* KO mutant mice infected with *C. difficile* showed enhanced virulence and IL1-β secretion and defective pathogen clearance and neutrophil recruitment. The mutant mice had impaired induction of cytokines/chemokines such as CXCL1. The data suggested that NOD1-mediated neutrophil recruitment is important for innate defense against the enteric pathobionts.^93^ Further studies have shown that the MyD88 adaptor protein, which is activated via TLR signaling, plays key role in neutrophil recruitment through expression of CXCL1 in *C. difficile*-mediated colitis.^94^ Additionally, leptin receptors have been shown to play role CDI-mediated colitis.^95^ Genetic polymorphism influences the severity of the inflammation because of the LEPR Q to R mutation in the leptin receptor in humans and mice.^95^ As compared to the QQ/QR genotype, the RR mice had exaggerated neutrophilia, increased inflammatory cytokines, and severe damage to the colon as well as increased expression of the CXC chemokine receptor 2 (CXCR2).^95^

Tryptophan metabolism by *C. difficile* also influences the outcome of CDI pathology and the neutrophil response. The enzyme IDO1 (indoleamine 2,3-dioxygenase-1) mediates the tryptophan metabolism and catalyzes the conversion of tryptophan to kynurenine and other metabolites. Inhibition of tryptophan catabolism in *ido1*-/- KO mice has been shown to result in severe CDI-mediated mucosal destruction and activation of neutrophils to produce proinflammatory cytokine IFN-γ. Additionally, kynurenine-induced apoptosis in bone marrow-derived neutrophils while tryptophan ameliorated the effect.^96^ Therefore, tryptophan metabolism seems to play a protective role against CDI. Treatment supplementation with the kynurenine may provide another therapy against CDI.

Activation of the P2Y6 receptor (a G-protein coupled receptor) in Caco-2 cells, which releases UDP (uridine diphosphate) as a danger signal, has been linked to the induction of CXCL8/IL-8, a potent neutrophil chemoattractant following the exposure to TcdA and TcdB.^97^ Treatment with the P2Y6-receptor-antagonist MRS2578 abrogated the toxin-induced CXCL8/IL-8 release and inflammation in mice.^97^ These data open new avenues for treatment of severe inflammation in CDI mediated colitis.

### Activation of Th17 response

Th17 cells with transcriptional regulator factor RORγt are subsets of CD4^+^ T cells that have been reported to be involved in autoimmune diseases and inflammatory diseases such as IBD and colitis with elevated expression of IL-17.^98^ The important inflammatory signature cytokines secreted by these cells are IL-17A and IL-17 F. These cells also produce TNF-α and IL-22. Th17 cells that produce anti-inflammatory cytokines IL-10 in the intestines, also known as Regulatory Th17 cells, have been reported to be transdifferentiated from Th17 cells.^99^ The role of IL-17 in neutrophil recruitment have been established in many studies. IL-17 A and IL-17 F double knockout (IL-17 KO) mice with decreased neutrophil infiltration in the colon have been shown to be more resistant to CDI than the wild-type mice.^100^

Using dextran sulfate sodium-induced colitis, Saleh *et al.^9^* showed that colitis induces Th17 cells that are still available in lymph nodes after recovery from the colitis. Adoptive transfer of these Th17 cells increased the mortality and severity of the disease after challenge with *C. difficile.^9^* However, unlike adults, young children are highly resistant and show protection against CDI.^101^ Chen e*t al*.^101^ examined the role of IL-17A produced by γδ T cells in CDI and showed the presence of elevated levels of IL-17A and T cell receptor γ chain expression in stool samples from children. The authors also demonstrated that in neonatal mice the presence of RORγt^+^ γδ T cells, which produce IL-17, were resistant to CDI while the depletion of these cells abrogated the protective effect.^101^ Markey *et al*.^102^ showed that pre-colonization with commensal *Candida albicans* protects against lethal CDI. The mice pre-colonized with *C. albicans* expressed elevated levels of IL-17A following *C. difficile* challenge as compared to the non-colonized mice. Additional administration of IL-17A to the pre-colonized mice offered protection against the CDI.^102^ Therefore, the IL-17 may have a dual detrimental or protective role depending on the source of the immune cells and types.

## Protective immune responses against pathobiont assault

Upon infection, the enteric pathobiont *C. difficile* inflicts damage to the intestinal epithelium, causing increased permeability, which helps the pathobiont further invade into the deeper tissues. Host resident cells that participate in the protection against the pathobiont are activated and a rapid innate response is mounted by induction of pro-inflammatory cytokines/chemokines and reactive oxygen and nitrogen species, which help recruit the innate cells to the site of infection followed by the adaptive cell response. A prolonged inflammatory response proves detrimental to the host as it causes damages to the epithelial barrier. Although reactive oxygen species cause much damage to the cells, *C. difficile* is relatively resistant to this episode. Reactive nitrogen species inhibit the toxin activity by S-nitrosylation of TcdA and TcdB at cysteine protease domain and thus provide protection from the CDI.^103^ Involvement of a cellular response in terms of T cells and B cells has not proven effective as the Rag1-/- KO mice lacking T cells and B cells recover from acute infections like the wild-type mice.^104,105^

A recent study^106^ using the mouse model of CDI showed a poor primary antibody response and an inadequate B memory response that does not protect in the recurrent infection model. In addition, there was a poor proliferation of T follicular cells. However, immunization of these mice generated B-memory cells as well as plasma cells.^106^ In CDI patients, adaptive immunity has been shown to provide protection as evidenced by the clinical data.^107^ The presence of IgA and IgG antibodies against *C. difficile* toxins and treatment with toxin-specific humanized monoclonal antibodies show protection from recurrent infections.^107^ Infants and toddlers are resistant to *C. difficile*-mediated colitis, although almost half are colonized by C. difficile.^101^ Thus, *C. difficile* resides as a normal constituent of the gut microbiome from childhood on and later in life becomes a pathobiont. It has been shown^108^ with a cohort of healthy infant population that toxigenic *C. difficile* colonization was accompanied with higher serum antitoxin IgA and IgG titer against TcdA and TcdB. Furthermore, there were significant neutralizing antibody (NAb) titers against TcdB. The protective humoral response associated with *C. difficile* colonization in infants further confirmed the resistance mechanism in infant population.^108^

Innate response to the pathobiont is critical for host survival as innate cells like neutrophils play crucial roles in the pathogenesis of the CDI. In addition to neutrophils, Buonomo *et al*.^109^ reported that eosinophils provide protection against the *C. difficile* pathobiont in an IL-25-dependent manner. Recombinant IL-25-treated mice showed less severe disease phenotype that was associated with decreased epithelial barrier damage and elevated levels of IL-4.^109^ Eosinophil-deficient mice showed severe epithelial damage and increased colitis, which shows the protective role of eosinophils.^109^ Another study,^110^ using the toxin transferase in *C. difficile* R20291-induced severe colitis, showed apoptosis of eosinophils. Since the intestinal IL-25 expression is suppressed in CDI and restoration with recombinant IL-25 reverses the disease phenotype, IL-25 therapy may provide a therapeutic advantage for disease severity. However, how eosinophils confer protection is largely an unknown mechanism. Further detailed mechanistic investigation may open new avenues for CDI treatment.

### Role of Innate Lymphoid Cells (ILCs) in protection

Innate lymphoid cell type 3 (ILC3s) along with CD4^+^ Th17 cells and CD8^+^ Tc17 cells induce type 3 immune responses and have been implicated in IBD and CDI.^104,111^ ILCs are polarized cell types of CD4 + T helper (Th) cells, which resemble Th1, Th2, and Th17 cell types but lack antigen specificity. ILCs mount a rapid immune response to restrict the pathogen invasion. Based on their transcription factor markers and signature cytokines, these cells have been grouped as three major cell types; ILC1 are T-bet^+^ and secrete IFN-γ and TNF-α; ILC2 are GATA3^+^ and secrete IL-5, IL-9, and IL-13; ILC3 are Rorγt^+^ and secrete IL-22, IL-17, TNF-α, and GM-CSF.^112^

The function of transcription factor Nfil3, which has a key role in the developmental process of ILCs (Figure 2), has been investigated in terms of protection against the enteric pathogen *Citrobacter rodentium* and *C. difficile* in Nfil3-deficient mice.^111^ It was demonstrated that numbers of ILC3s were significantly reduced in the *nfil3*-/- KO mice. Nfil3 was indispensable for the development of the gut associated ILC3s (Figure 2). Nfil3-deficient mice were more prone to develop acute infections caused by the gut pathobionts *Citrobacter rodentium* and *C. difficile*.^111^ A subsequent study^104^ investigated the role of ILCs in Rag1-/- deficient mice that lacked T and B cells and *ragγc*-/- mice that lacked ILCs. The ILC-deficient mice were susceptible to infection and quickly succumbed to infection as compared to the Rag1-/- mice.^104^ Protection was restored by transfer of ILCs into RagγC-/-KO mice. The study^104^ demonstrated that the ILC1 were critical for host defense. The RagγC-/- KO mice that were defective in IFN-γ or T-bet^+^ cells were more susceptible to CDI.

The potential immune targets that are crucial in protection and quick recovery was investigated further using transcriptomic analysis.^113^ Frisbee *et al*.^113^ identified the cytokine IL-33, a member of IL-1 cytokine family that was important for gut barrier function during colitis and was upregulated during CDI. The IL-33 plays key role in activating ILC2s, which prevented the *C. difficile*-induced colitis in the mouse model (Figure 2).^113^ Furthermore, the study predicted that the dysregulation of IL-33 signaling via the decoy receptor sST2 in human CDI is associated with mortality.^113^ Frisbee *et al*.^113^ also discussed the role of microbiota in the induction of IL-33 response as previous studies in germ-free murine IBD models detected reduced expression of IL-33 in chronic ileitis of the small intestine. Treatment with murine FMT and spore-based fecal preparation from human source could rescue colonic IL-33 in mice.^113^ The microbiota-dependent upregulation of IL-33 needs further investigation for *C. difficile*-induced infections.

Fachi *et al*.^47^ demonstrated the role of microbiota-derived acetate in protection through the recruitment of neutrophils and ILC3s in acute mouse model of CDI. Acetate signals through free fatty acid receptor 2 on neutrophils and ILC3s and accelerates their recruitment to the site of infection. Acetate–FFAR2 interaction activated the inflammasome that resulted in the release of IL-1β and further promoted the secretion of IL-22 in ILC3 through IL-1 receptor signaling in response to IL-1β (Figure 2).^47^ In the mouse CDI model, secretion of IL-22 cytokine was induced and the IL-22-/- KO mice showed increased pathobiont load and resistance to complement mediated phagocytosis.^105^ Administration of IL-22 helped clear the pathobiont load by increased phagocytosis.

### Induction of protective regulatory T cells response

Pathological conditions leading to inflammatory diseases such as IBD and pseudomembranous colitis are associated with dysregulation of immune homeostasis. Increasing evidence using mouse models suggests the key role of regulatory T-cells (T_reg_) in the protection and dysregulation by microbiota-associated pathobionts leading to diseased condition.^114^ T_reg_ cells have suppressive activity toward an exacerbated immune response in an inflammatory condition and inhibit the response of effector cells. Peripherally induced T_regs_ (pT_regs_) are a subset of CD4+ T cells that express the Foxp3+ CD4+ CD25+ marker. They are induced by the transforming growth factor β (TGF-β). In the gut-associated lymphoid tissues, these naïve CD4+ T cells, upon interaction with antigen are differentiated to pT_reg_ in the presence of TGF-β, IL-2, and retinoic acid, which are produced by microbiota-conditioned CD103^+^ dendritic cells.^114^ In contrast, CD103-dendritic cells (DCs) do not induce T_reg_ cells. Microbiota-conditioned CD103^+^ dendritic cells in IBD patients fail to induce pT_reg_ cells, but instead induce the pathogenic Th1, Th2, and Th17 response. These findings suggest the role of pathobiont in tweaking the protective role to pathogenic condition in IBD.^115^

Due to encounters with microbial antigens in the intestinal lamina propria, pT_regs_ provide tolerance by co-expressing the RORγt transcription factor, which is also expressed on pathogenic Th17 cells. Foxp3+ RORγt+ pT_regs_ have been shown to provide protection and tolerance against the gut microbiota (Figure 2).^115^ When these cells express GATA3, a Th2 transcription factor facilitates the repair of the damaged intestinal mucosa. Furthermore, GATA3^+^ T_regs_ express the IL-33 receptor (ST2) and an epidermal growth factor receptor, amphiregulin.^116,117^ The presence of the IL-33 receptor in T_reg_ cells is important because, as discussed earlier, the IL-33 receptor induces a protective response in CDI. T_reg_ cells maintain intestinal equilibrium by secretion of the anti-inflammatory IL-10 cytokine. The IL-10-/- KO mice have been shown to be particularly susceptible to developing colitis.^118^

The transcription factor c-Maf that is highly expressed in intestinal T_reg_ is a critical regulator of IL-10. Furthermore, c-Maf is essential for differentiation of RORγt-expressing T_reg_ cells in response to pathobionts (Figure 2).^35^ Wheaton *et* *al.*^119^ suggested the key role of c-Maf in the regulation of intestinal T_reg_ cells for host microbiota homeostasis. The differentiation of naïve T cells into RORγt +Foxp3+ T_reg_ cells in response to the pathobiont *H. hepaticus* was c-Maf dependent. In the absence of c-Maf, the naïve T cells differentiated to Foxp3-TH17 cells that caused intestinal inflammation.^119^ Subsequently, it was established that c-Maf is absolutely required for the induction of T_reg_ cells to control the development of microbiota-dependent IgA and Th17 cells in the gut.^120^ Furthermore, c-Maf controlled the IL-10 production in T_reg_ cells. Deficiency of c-Maf caused dysbiosis in the gut and enhanced expression of TH17 cells.^120^ Therefore, the study underpins the importance of c-Maf dependent function of T_reg_ cells in maintaining gut homeostasis.

## Identification and quantification of pathobionts

**Figure 3.**
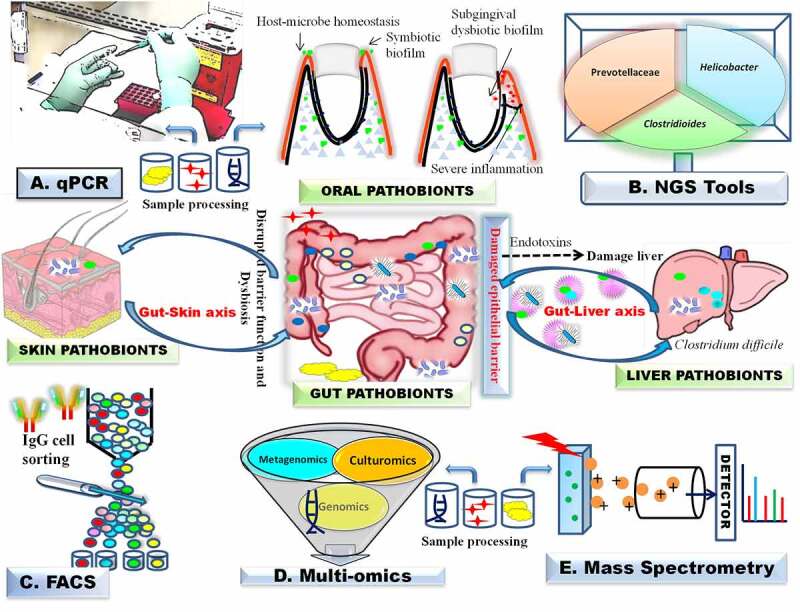
**Pathobiont detection methods** (A-E). A. Biopsy and fecal samples are used for DNA isolation, followed by PCR amplification; B-E. FACS, MALDI-ToF and multi-omics technologies such as genomics, transcriptomics proteomics, metabolomics, and microbiomics are used for real time monitoring of pathobionts and their metabolites. Dysbiotic pathobiont promote bacterial translocation to liver via intestinal epithelial cell barrier dysfunction and mesenteric lymph nodes. FACS, fluorescence-activated cell sorting. Illustrations were made using Biorender tool

Fast diagnosis is critical for treating pathobiont infection and disease management, including CDI. Laboratory diagnostic assays for CDI such as cell cytotoxicity assay, glutamate dehydrogenase test, enzyme immunoassays, and tests based on nucleic acid sequences have limitations in rapidity, sensitivity, and specificity. They may give inconsistent false-positive or false-negative results, although positive and negative references are always included in sample sets.^121^ As illustrated in Figure 3, various tools and techniques are used to study the diversity and host–pathobiont interactions for pathogens isolated from the host microbiome. The phenotypes, i.e., adhesion, invasion, and survival of the pathobionts, are studied by the macrophage assays. Pathobiont strains have been found to survive within macrophages at significantly higher rates than non-pathobionts due to an interconnection between the production of phagosomal reactive oxygen species by the macrophages.^122^

The abundance, virulence factors, genotyping, and antimicrobial susceptibility testing (antibiogram profile) of the pathobionts can be determined by techniques like VITEK® 2 for automated microbial identification and antibiotic susceptibility testing, pulsed-field gel electrophoresis, and nuclei acid amplification by polymerase-chain reaction (PCR, RT-PCR, and qPCR).^123–125^ None of the techniques alone can however provide comprehensive information needed to quantify and study the multiplicity of appearance, effect, and pathosis of pathobionts.

The virulence genes *chu*A and *rat*A were isolated from IBD patients and were used to differentiate between pathobiontic AIEC strains and non-AIEC strains.^124^ Generally, the PCR amplification followed by restriction fragment length polymorphism (RFLP) analysis is used for the better resolution of several clinical serotypes of other pathobionts.^126^ The RFLP method is versatile, discriminative, and has resolution in studying specific strains, but it lacks broad applicability and microbial quantification. The qPCR techniques are routinely used to determine the number of gene copies of pathobionts in clinical samples.^127^

The MALDI-TOF (Matrix-Assisted Laser Desorption/Ionization Time-of-Flight) MS and other mass spectrometry strategies such as rapid evaporative ionization mass spectrometry (REIMS) contribute significantly to the development of real-time methods for the chemotaxonomic classification of microbiota including pathobionts at the genus and species levels. MALDI-TOF MS or ESI-TOF (Electrospray Ionization Time-of-Flight) technology that depends upon the reference database of enriched peptide mass fingerprints database of the type strains can be used to identify pathogens from prepared clinical samples, i.e., stools and urine, to overcome the limitations of the cultivation methods.^128^ Microbial identification with the help of meta-proteomic data is based on the analysis of unique, strain-specific peptide biomarkers, which are also examples of noninvasive monitoring of pathobionts at the genus and species levels.^128^ REIMS is a high-throughput MS-based method that is used in the metabolomics and metabolite profiling of a wide range of human samples including cell culture, and fecal microbiota.^129^ During a *C. difficile* outbreak, S-layer proteins (30–50 kDa region) were diagnosed using the MALDI-TOF MS method.^130^ The technology can be used in combination with PCR ribotyping, toxinotyping, and next-generation sequencing data to improve epidemiological studies.

### Next generation sequencing technologies

Multi-omics are high-throughput scale-up technology that have revolutionized research in human microbiome, comprising commensals, symbionts, and pathobionts. The multi-omic technologies, such as genomics, transcriptomics proteomics, metabolomics, and microbiomics, are now often used to explore human mucosal surfaces, skin, and the gut that are colonized by fungi, bacteria, and viruses, collectively known as the microbiota.^131^ Next-generation sequencing technologies have been continuously evolving over the past 15 years, leading to substantial improvements in both yield and quality. They are now in routine use for sequencing DNA and RNA of clinical samples, real-time monitoring of pathobionts, and massive human genome and gut microbiome projects.^131^

The advancement in the next-generation sequencing technology has substantially increased the reporting of pathobionts from the human gut, and together with metagenomics, it has evolved as an indispensable tool in this multi-omics era. Human gut metagenome studies have revealed probable links between the gut microbiome and rheumatoid arthritis, depression, obesity, and diabetes. In a recent study,^132^ DNA samples from vaginal swabs were amplified for the 16S rRNA gene (V3–V4 variable regions) and sequenced on Illumina platform. The sequence data revealed lactobacillus-dominated genera, and 40 pathobionts, dominated by six non-minority taxa, i.e., *Streptococcus* (54% of pathobionts reads), *Staphylococcus, Campylobacter, Enterococcus, Haemophilus, Escherichia*, and *Shigella*.^119,132^ The results suggest that pathobionts co-occur with lactobacilli and bacterial vaginosis (BV) anaerobes. The meta-analysis of the participant vaginal microbiota revealed the presence and levels of the pathobionts and their negative correlations between relative abundances (ρ = −0.9234) with lactobacilli, and positive correlation (r = 0.1938) with BV-anaerobes, and their associations with behavioral, socio-demographic, and clinical attributes. These results were a powerful demonstration of molecular analytical techniques providing detailed insights in microbial diversity and associations at genomic levels. Clearly, interpretation of pathobionts and microbiota in clinical studies can be enriched with multiple analytical methods.

The genome sequencing of *C. difficile* strains along with the epidemiological data are extremely useful to understand the transmission clusters during epidemic situations.^133^ A recent report on the relatedness in the genomes of successive *C. difficile* isolates (difference of ≤ 2 single nucleotide polymorphisms) revealed 17 independent clusters, which were previously undetected and unreported by the standard epidemiological surveillance tools.^133^

In bacterial lower respiratory infections, clinical investigation is a benchmark technique; however, it is time-consuming and less sensitive than molecular methods. A cost-competitive and advanced technique like the real-time nanopore metagenome sequencing can be used for rapid and accurate characterization of human pathobionts, including pathogens present in lower respiratory infections.^134^ Conclusively, the critical evaluation of the tools and techniques used in the study of human microbiome suggests that different pathogens including pathobionts need a combinatorial approach for detailed, in-depth understanding of their phylogeny and ecological and metabolic functions. Deep information obtained from whole-genome sequencing data along with amplification and analysis of genes will be useful in the diagnosis of CDI, strain detection, genetic diversity, and epidemiology. It is anticipated that mRNA-based analyses of gene expression and protein synthesis, now routine in molecular microbial physiology and ecology, will be employed increasingly in molecular diagnostics of disease manifestation and underlying analysis of toxigenic microbial biomolecules.

## Conclusions and future perspectives

The human microbiome with its vast pool of genes performs a key role in the overall health of the host and maintains a homeostasis in the gut by interaction with the mucosal immune system. This intricate interaction is critical for epithelial barrier function, which helps sensitization of the mucosal innate immune cells for tolerance toward the commensals. However, under unfavorable environmental conditions or genetic alterations, the dynamic homeostasis is disrupted leading to dysbiosis and emergence of pathobionts. Exposure of pathobionts is detrimental to the host epithelial barrier due to induction of exacerbated innate response leading to severe inflammatory conditions such as pseudomembranous colitis and IBD. The emergence and survival of *C. difficile* needs further investigation in terms of key transcription factors that regulate key steps of germination, biofilm formation, and sporulation in host in relation to the toxin production. Identification of the target genes of these transcription factors will facilitate a clear understanding of the pathogenicity of this pathobiont.

Recent groundbreaking studies have shed more light on the emergence of pathobionts and the host mediated protection mechanisms by immune cells such as _Treg _ cells, Th17 cells, and innate lymphoid cells. Further identification of pathobionts-related antigens that induce protective mechanisms might help development of a novel vaccine against pathobionts.

The detection and quantification of pathobionts have been greatly facilitated by cutting-edge technologies, giving much-needed insight in the pathobiont research. A combination of both immunological markers and the proteomics tool will further aid in correctly identifying the pathobiont and the target molecules. A clear understanding of the key factors and biomolecules involved in the emergence of pathobionts and the virulence factors that mediate inflammation are potential targets for development of novel therapeutics and personalized medicine for these severe chronic inflammatory conditions.
